# Age-Related Memory Impairment Is Associated with Disrupted Multivariate Epigenetic Coordination in the Hippocampus

**DOI:** 10.1371/journal.pone.0033249

**Published:** 2012-03-15

**Authors:** James F. Castellano, Bonnie R. Fletcher, Bennett Kelley-Bell, David H. Kim, Michela Gallagher, Peter R. Rapp

**Affiliations:** 1 Laboratory of Experimental Gerontology, National Institute on Aging, Baltimore, Maryland, United States of America; 2 Graduate Program in Neuroscience, Mount Sinai School of Medicine, New York, New York, United States of America; 3 Department of Psychological and Brain Sciences, Johns Hopkins University, Baltimore, Maryland, United States of America; Pontifical Catholic University of Rio Grande, Brazil

## Abstract

Mounting evidence linking epigenetic regulation to memory-related synaptic plasticity raises the possibility that altered chromatin modification dynamics might contribute to age-dependent cognitive decline. Here we show that the coordinated orchestration of both baseline and experience-dependent epigenetic regulation seen in the young adult hippocampus is lost in association with cognitive aging. Using a well-characterized rat model that reliably distinguishes aged individuals with significant memory impairment from others with normal memory, no single epigenetic mark or experience-dependent modification in the hippocampus uniquely predicted differences in the cognitive outcome of aging. The results instead point to a multivariate pattern in which modification-specific, bidirectional chromatin regulation is dependent on recent behavioral experience, chronological age, cognitive status, and hippocampal region. Whereas many epigenetic signatures were coupled with memory capacity among young adults and aged rats with preserved cognitive function, such associations were absent among aged rats with deficits in hippocampal memory. By comparison with the emphasis in current preclinical translational research on promoting chromatin modifications permissive for gene expression, our findings suggest that optimally successful hippocampal aging may hinge instead on enabling coordinated control across the epigenetic landscape.

## Introduction

Epigenetic modifications support persistent cellular memory allowing terminally differentiated cells to sustain their phenotype. Recent evidence encourages the view that the nervous system co-opts these mechanisms in support of a variety of dynamic capacities including synaptic plasticity (for recent review see [1]). Multiple studies have linked increased histone acetylation to hippocampal memory, presumably reflecting the induction of chromatin modifications permissive for the transcription of learning-related plasticity genes [2], [3], [4], [5], [6]. The bidirectional control of histone acetylation is regulated by histone acetyltransferases [2] and histone deacetylases (HDACs), and these factors also have been shown to influence learning and memory [2], [7], [8], [9]. Prolonging histone acetylation pharmacologically with HDAC inhibitor administration, for example, increases synaptic connectivity in the hippocampus, enhances LTP, and benefits memory [4], [10], [11], [12], [13], [14].

Cognitive impairment associated with advanced chronological age is a well-documented outcome across multiple species, including rodents, monkeys and humans [15], [16]. Many features of neuronal integrity remain intact in aged rats with deficits in hippocampus dependent memory [17], [18], [19]. Instead of diffuse deterioration, normal cognitive aging is associated with a constellation of subtle and neuroanatomically specific alterations involving intracellular signaling pathways, gene expression, and other memory-related plasticity mechanisms [15], [20], [21], [22]. Alongside rapid progress in many other areas of neuroscience, including addiction [23], [24], stress [25], [26], and neurological disease (for review see [27]), less attention has focused on identifying potential epigenetic contributions to normal cognitive aging (for exceptions see [5], [28]). Among the significant challenges in this area, it has proved difficult to distinguish the epigenetic consequences of chronological aging from changes that might specifically contribute to differential cognitive outcomes.

Using a well-established model of age-related learning and memory impairment [29], here we examined levels of histone acetylation, HDACs, and a protein with intrinsic HAT activity in the hippocampus of young (Y), cognitively intact (aged unimpaired; AU), and cognitively impaired (aged impaired; AI) aged rats. A key feature of this model is that it reliably discriminates aged individuals in relation to differences in hippocampal integrity. The principal fields of the hippocampus mediate partly dissociable processing functions in support of normal memory, and considerable evidence points to the differential vulnerability of these subfields to a variety of conditions, including aging [30]. The hippocampus was therefore microdissected in our analysis, testing the possibility that the epigenetic consequences of aging and recent experience are regionally selective across the dentate gyrus, CA3 and CA1. In order to examine both resting and dynamic chromatin regulation we compared markers examined in Y, AU, and AI animals provided recent behavioral experience (i.e., histone acetylation, HDAC, and HAT levels) with results from age- and cognitive status-matched rats sacrificed directly from the home cage. This design allowed tests of potential constitutive, experience-dependent, and interactive effects of aging on epigenetic control in the hippocampus.

## Results

### Spatial learning reveals substantial individual differences in the cognitive effects of aging

**Figure 1:**
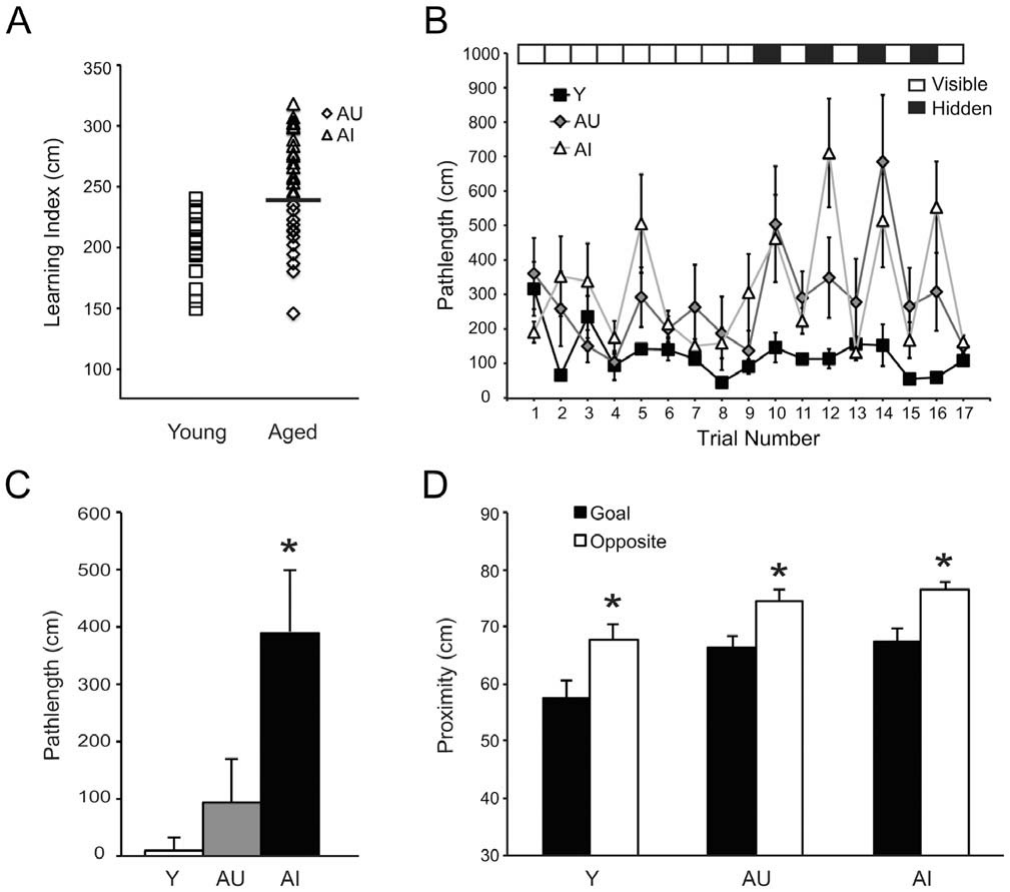
A subset of aged Long-Evans rats displays impaired spatial memory. (A) Learning index scores (a weighted measure of proximity to the platform location across interpolated probe trials) for individual young and aged rats in a standardized spatial version of the Morris water maze. Aged rats (24 mos) that scored within the range of young (Y; 6 mos) were classified as aged unimpaired (AU) and the remainder were classified as aged impaired (AI). (B) Redundant place/cue (RPC) task schematic (top), and escape performance during training, measured as pathlength, in Y, AU, and AI subjects. (C) AI animals displayed impaired spatial learning in the RPC task relative to Y and AU animals, measured as the difference in average pathlength between the last four hidden and visible trials. (D) All groups showed significant spatial bias, measured as proximity to the goal location, during a probe trial provided two hours after the onset of RPC training. Error bars = S.E.M; asterisks indicate p-values<0.05.

Specific pathogen-free young (6 mos) and aged (24 mos) male Long-Evans rats were characterized in a standard place version of the Morris water maze [29], [31]. Consistent with previous studies [17], [18], [19], approximately half of the aged subjects displayed deficits relative to young rats, whereas the other half performed within the range of adult controls (Fig. 1A). By comparison with earlier studies where aging was uniformly associated with impairment [5], [28], the individual differences observed here provided a framework for evaluating the cognitive outcome of aging in relation to chromatin regulation.

Approximately one month after initial water maze characterization, a single, relatively brief session of redundant place/cue (RPC) training was provided as an inducing event for examining dynamic chromatin modification. With a visible escape platform held in a constant location, testing consisted of 9 cued trials, followed by 8 alternating cued and non-cued (hidden platform) trials. Testing with a cued, fixed escape location is amenable to both goal-approach and allocentric spatial solutions, whereas efficient escape to a hidden platform requires memory for extra-maze spatial information [32]. Thus, the design allowed for either hippocampus dependent or independent task strategies while matching relevant performance variables (e.g., sensorimotor demands) across young and aged rats. All groups showed rapid acquisition, displaying progressively shorter pathlengths to the cued escape platform across the first 9 trials (main effect of trial, F_(8,192)_ = 2.88, p = .005; Fig. 1B). Spatial learning was evaluated as the average difference in pathlength between the 4 hidden and final 4 cued platform trials. By this measure values approaching zero reflect substantial transfer across trial types, and thus better memory for the escape location. AI animals scored significantly worse than Y (p<.005) and AU (p<.05) rats (main effect of group, F_(2,23)_ = 8.08, p<.005; Fig. 1C), whereas performance in the latter groups was statistically equivalent. Despite differences during acquisition, all groups showed significant spatial memory on a probe test provided two hours after the onset of training and immediately before sacrifice. On this trial the platform was unavailable for escape and spatial bias was measured as proximity to the goal location relative to an equivalent area centered in the opposite quadrant of the maze (Y, t_(1,15)_ = −2.858, p<.05; AU, t_(1,12)_ = −2.413, p<.05; AI, t_(1,18)_ = −3.420, p<.005; Fig. 1D).

### Basal and experience-dependent histone acetylation levels are regulated in relation to age and hippocampal region

Prompted by evidence linking histone acetylation to memory, we utilized quantitative western blotting methods to investigate experience-, age-, and hippocampal subregion-dependent histone post-translational modification (PTM) in our model of normal cognitive aging. Y, AU and AI rats sacrificed directly from the home cage provided baseline data. The aim here was to examine the general capacity for experience-dependent chromatin modification. Guided by previous studies of this sort, multiple modifications on histone H3 and H4 were selected for analysis: H3-pan-acetyl, H4-pan-acetyl, H3-acetylK9, and H3-phosphoS10.

**Figure 2:**
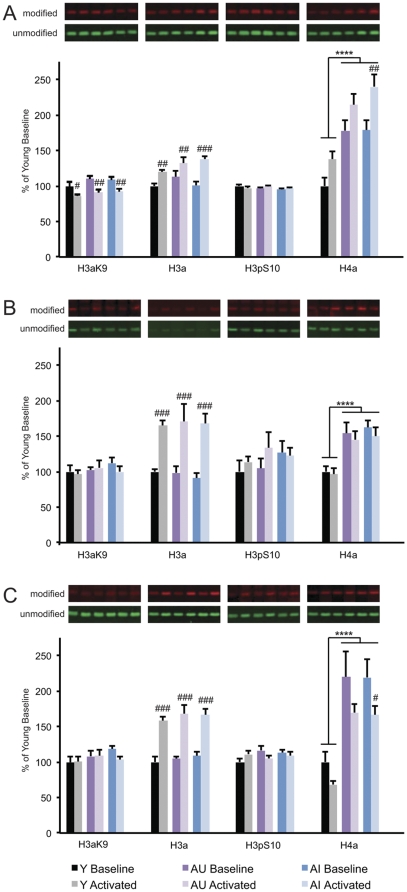
Histone PTMs are regulated in relation to age, recent experience and hippocampal region. (A–C) Quantification of histone PTM regulation in hippocampal regions CA1 (A), CA3 (B), and DG (C) in Y, AU and AI rats with (Activated) or without (Baseline) immediately preceding RPC training. Representative western blots are organized according to the corresponding quantitative results. Histone modifications were normalized to pan-histone H3 and group data are represented as the percentage of young baseline values. Error bars = S.E.M. Number signs and asterisks denote statistically significant experience- and age-dependent effects, respectively (p<0.05).

Significant histone acetylation effects observed in the present analysis prominently included experience-dependent regulation. These findings complement studies using contextual fear conditioning [4], [5], demonstrating that the modulation of histone acetylation extends to multiple behavioral settings designed to examine hippocampal memory. Here, histone H3 acetylation was potently increased in response to recent water maze experience across all hippocampal subregions (main effect of training for CA1, F_(1, 37)_ = 45.19, p<0.001; CA3, F_(1,38)_ = 46.19, p<0.001; DG, F_(1,40)_ = 89.99, p<0.001; Fig. 2). Whereas H3-pan-acetyl levels displayed a subfield-independent pattern of behavioral regulation, H3-acetylK9 and H4-pan-acetyl levels responded to activation differentially across the principal fields of the hippocampus. Specifically, lysine 9 acetylation on histone H3 was significantly decreased in response to recent behavioral experience in region CA1 (main effect of training F_(1,37)_ = 22.79, p<0.001; Fig. 2A), with no activation effects in CA3 (Fig. 2B) or DG (Fig. 2C). H4 acetylation, by comparison, was regulated in opposite directions in regions CA1 and DG; levels were increased in CA1 and decreased in DG (main effect of training for CA1, F_(1,37)_ = 10.79, p = 0.002; DG, F_(1,40)_ = 10.22, p = 0.003; Fig. 2A and C), with no effect of training observed in CA3 (Fig. 2B). This pattern of subfield-specific, bi-directional modification indicates that experience-dependent histone acetylation in the hippocampus is regulated with greater neuroanatomical resolution than previously recognized.

In addition to experience-dependent regulation, histone acetylation was significantly modulated in relation to chronological aging. Age effects were selective for histone H4, where acetylation levels were substantially greater in aged animals (both AU and AI) relative to young across all hippocampal subfields (main effect of age for CA1, F_(1,41)_ = 31.48, p<0.001; CA3, F_(1,42)_ = 38.17, p<0.001; DG, F_(1,44)_ = 47.98, p<0.001; Fig. 2). The observation that the epigenetic consequences of normal aging include sharply elevated histone acetylation levels in hippocampus is striking against a backdrop of reports that increased acetylation is broadly associated with enhanced memory [33]. Alongside this shifted baseline, aged animals retained the capacity for behavioral regulation, displaying a pattern of experience-dependent histone H4 acetylation qualitatively similar to young subjects (Fig. 2A, C). Together the findings reveal prominent influences of chronological age and recent behavioral experience, but suggest that no specific histone acetylation effect uniquely distinguishes aged rats with hippocampal cognitive impairment from young and aged rats with normal memory.

Next we examined phosphorylation of serine 10 on histone H3 (H3pS10), prompted by findings that this modification is also regulated by recent experience [34], [35], [36]. In contrast to histone acetylation, overall H3pS10 levels measured by western blotting appeared unaffected in relation to age, cognitive impairment, and behavioral training (Fig. 2). Immunocytochemical labeling, however, revealed a pattern of anatomical localization distinct from the acetyl-histone marks (Fig. S1). Whereas essentially all principal neurons in the hippocampus were highly immunopositive for acetyl modifications, only a small subset of dentate gyrus granule cells displayed intense phosphoS10 labeling (Fig. S1A). In separate cohorts of young animals we quantified by stereological methods the effect of RPC training on the number of these H3pS10 immunoreactive cells in DG. In contrast to the negative results obtained by western blotting, RPC training robustly increased the numbers of H3pS10 immunolabeled granule neurons relative to home cage control values (training effect for DG top blade t_(10)_ = −12.40, p<0.001; DG bottom blade t_(10)_ = −4.05, p = 0.002; Fig. S1B). These findings suggest that methods providing cellular resolution may prove important for a comprehensive accounting of epigenetic contributions to normal memory and age-related impairment.

### Bidirectional modulators of histone acetylation are regulated in normal cognitive aging

**Figure 3:**
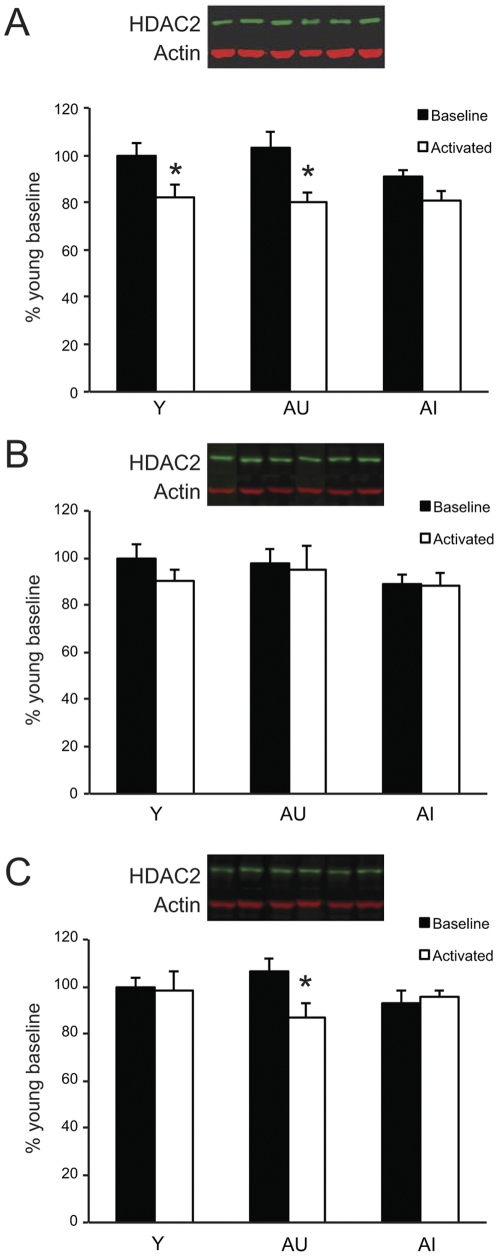
HDAC2 is regulated in relation to cognitive aging, recent experience and hippocampal region. (A–C) Quantification of HDAC2 levels in regions CA1 (A), CA3 (B) and DG (C). Representative western blots are organized according to the corresponding quantitative results. Protein levels were normalized to β-actin and the group data are represented as a percentage of young baseline values. Error bars = S.E.M. Asterisks denote p<0.05 relative to respective baseline.

Having documented that multiple histone PTMs are regulated in relation to behavioral training and age, next we considered enzymes critical for the bidirectional regulation of histone acetylation, i.e., HDACs and HATs. Substantial interest has centered on HDAC2 because this deacetylase, but not the structurally homologous HDAC1, reportedly regulates memory [7]. Here we found no change in baseline HDAC2 protein levels as a function of age or cognitive status in any hippocampal region (main effect of group for CA1, F_(2,17)_ = 1.774, p = .200; CA3, F_(2,17)_ = 1.392, p = .276; DG, F_(2,17)_ = 1.764, p = .201). In correspondence with the increase in histone acetylation seen for a number of marks subsequent to recent behavioral experience (Fig. 2), however, RPC training was associated with regionally selective decreases in HDAC2 protein levels (Fig. 3). Notably, this effect was observed selectively among cognitively intact animals such that in CA1, only Y and AU subjects displayed significant downregulation with training relative to age-matched, baseline controls (Y, t_(1,13)_ = 2.183, p<.05; AU, t_(1,11)_ = 2.994, p<.05; Fig. 3A). No experience-dependent change was observed in the CA3 field (Fig. 3B), whereas in DG, AU rats alone exhibited significant downregulation (t_(1,11)_ = 2.390, p<.05) (Fig. 3C), perhaps reflecting a compensatory response. These findings implicate HDAC2 in the epigenetic phenotype of cognitive aging.

Unlike HDAC2, HDAC1 protein levels were unaffected as a function of recent experience, age, and cognitive status. In conducting the Western blot analysis, however, we noted a distinct mobility shift of relevant bands for groups provided RPC training, suggesting that HDAC1 regulation in response to recent experience might involve post-translational protein modifications. We explored this by quantifying levels of HDAC1 phosphorylated at serine 421, i.e., a modification known to confer increased catalytic activity and affinity for co-repressor complexes [37]. Compatible with our histone acetylation and HDAC2 results, and with emerging evidence for activity-induced derepression of acetylation [38], behavioral training led to a substantial reduction in phospho-HDAC1 levels in all hippocampal subfields (main effect of training for CA1, F_(1,44)_ = 64.835, p<.005; CA3, F_(1,44)_ = 81.044, p<.005; DG, F_(1,43)_ = 108.781, p<.005). The magnitude of this effect, however, was unrelated to age and cognitive status (Fig. S2A).

HDAC1 and 2 are highly homologous proteins, and despite predicted functional redundancy, our findings corroborate previous data suggesting they play distinct roles in learning and memory [7]. We examined a possible basis for this dissociation using immunocytochemical labeling and confocal microscopy to document the cellular location of HDAC1 and 2 in brains from 9 home cage control rats (3 each for Y, AU and AI). Consistent with previous qualitative descriptions [7], [39], HDAC2 was localized to neuronal nuclei throughout the pyramidal and granule cell layers of the hippocampus. HDAC1 immunoreactivity, by comparison, was predominantly localized to GFAP-positive astrocytes (Fig. S3). Whereas CA1 pyramidal and granule cell nuclei also displayed HDAC1 signal, the nuclei of CA3 pyramidal neurons were devoid of staining. This cell-type selective expression pattern may help explain the distinct roles of these proteins in memory related plasticity and cognitive aging. Significant non-nuclear HDAC1 localization was also detected, underscoring that HDACs can deacetylate many non-histone proteins [40], [41].

HDACs oppose the influence of HATs on histone acetylation, and next we examined CBP, a transcriptional co-activator with intrinsic HAT activity implicated in learning and memory [9], [10]. A detailed analysis of constitutive, baseline levels of CBP will be presented elsewhere, documenting that this parameter is entirely unaffected as a function of age or cognitive status (Periera, et. al., Program No. 891.1, Society for Neuroscience, 2009. Online.). In the present experiments, behavioral training was associated with a robust reduction in CBP levels across all hippocampal subfields (main effect of training for CA1, F_(1,44)_ = 20.678, p<.005; CA3, F_(1,44)_ = 81.121, p<.005; DG, F_(1,41)_ = 37.595, p<.005; Fig. S2B). Few reports have documented experience-dependent CBP protein regulation (but see [2]), and while the direction of effect was unexpected, the precise temporal dynamics of bidirectional chromatin modification induced by experience remain to be fully characterized. It seems reasonable to suppose, for example, that CBP downregulation two hours post-training, as in the current experiments, may reflect negative feedback driven by earlier experience-dependent increases in histone acetylation.

### Cognitive aging uncouples coordinated epigenetic regulation from individual differences in spatial memory

**Figure 4:**
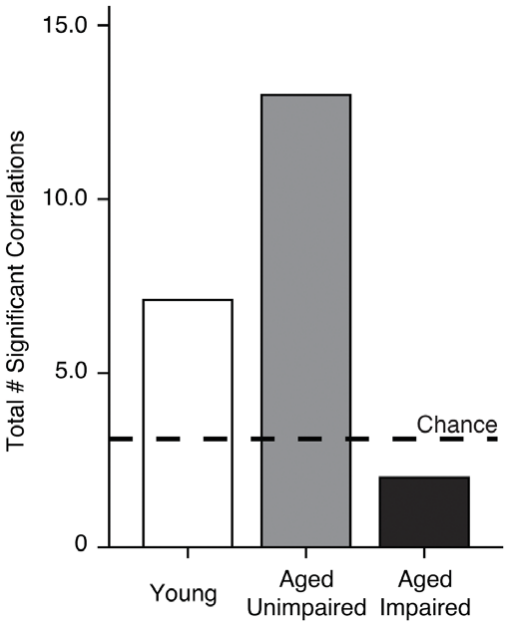
Total number of significant epigenetic/spatial memory correlations for Y, AU, and AI rats. Summary of results from Table 1, illustrating the number of statistically significant correlation coefficients observed between the epigenetic marks examined and learning index scores from the standard spatial version of the water maze. Only correlations computed for the Y, AU and AI groups considered separately were included, i.e., cases where sample size and within-group variance was similar. Chance reflects from the number of correlation coefficients computed per group (i.e., 63), and the nominal alpha value (i.e., 0.05). The number of significant correlations exceeded chance selectively for Y and AU groups.

In addition to allowing group comparisons, the aging model used here enabled a direct test of the proposal that patterns of chromatin regulation are coupled with individual differences in the integrity of spatial memory. Using learning index scores from the standard water maze characterization as a summary measure, we evaluated correlations between hippocampal histone PTMs, HDAC, and HAT levels, and spatial memory capacities. The results are listed in Tables 1 and 2, including Pearson r correlation coefficients and associated p-values for the Y, AU and AI groups considered separately and for the data collapsed across either all subjects (Y, AU and AI), cognitively intact animals (Y and AU), or aged rats (AU and AI; panel B). Whereas the number of statistically significant correlations greatly exceeded chance when either young or aged unimpaired rats were considered (italicized cells in Tables 1 and 2; columns Y, AU, All and Intact), the chromatin modification results were nearly universally unrelated to individual differences in spatial learning and memory among the aged subjects when impaired animals were included the analysis (Tables 1 and 2; columns AI, and Aged). Figure 4 summarizes the pattern seen when the Y, AU and AI groups were analyzed separately (data from Table 1). These findings indicate that, even when sample size and the spread of data are similar across groups, the total number of statistically reliable correlations between memory and epigenetic markers is substantially greater than expected by chance among Y and AU rats, but not in aged subjects with memory impairment. Beyond changes attributable to chronological age, the overall pattern points to substantial disruption in coordinated chromatin regulation in the hippocampus specifically in association with cognitive aging.

**Table 1:** Cognitive aging uncouples coordinated epigenetic regulation from individual differences in spatial memory.

Group		Y						AU						AI					
Region		CA1		CA3		DG		CA1		CA3		DG		CA1		CA3		DG	
		r	p	r	p	r	p	r	p	r	p	r	p	r	p	r	p	r	p
**H3aK9**	All	0.421	0.12	***−0.639***	***0.01***	−0.295	0.29	***0.578***	***0.04***	0.029	0.93	0.327	0.28	0.410	0.09	−0.062	0.81	−0.036	0.89
	Base	0.545	0.26	−0.621	0.19	−0.209	0.69	***0.983***	***<0.01***	0.138	0.79	−0.212	0.69	0.290	0.49	0.091	0.83	−0.087	0.84
	Act	0.089	0.82	***−0.833***	***0.01***	−0.356	0.35	0.033	0.94	0.104	0.83	0.695	0.08	0.592	0.07	−0.199	0.58	−0.082	0.82
**Hac**	All	−0.519	0.06	−0.181	0.52	−0.505	0.06	***−0.630***	***0.03***	−0.137	0.66	***−0.740***	***<0.01***	0.033	0.90	0.029	0.91	−0.230	0.36
	Base	−0.746	0.09	0.383	0.45	−0.481	0.34	−0.127	0.81	−0.345	0.50	−0.623	0.19	0.117	0.78	***−0.730***	***0.04***	−0.305	0.46
	Act	−0.181	0.67	0.496	0.17	−0.280	0.47	***−0.865***	***0.03***	0.569	0.18	−0.456	0.30	−0.043	0.91	0.406	0.25	−0.299	0.40
**H3pS10**	All	−0.284	0.31	***−0.683***	***<0.01***	−0.314	0.25	−0.503	0.08	−0.344	0.25	***0.590***	***0.03***	−0.286	0.25	−0.374	0.13	−0.211	0.40
	Base	−0.786	0.06	***−0.953***	***0.01***	−0.256	0.63	0.058	0.91	0.256	0.63	0.478	0.34	−0.607	0.11	−0.269	0.52	−0.051	0.91
	Act	−0.361	0.34	−0.593	0.09	−0.154	0.69	−0.620	0.14	−0.268	0.56	0.537	0.21	−0.156	0.67	−0.497	0.14	−0.326	0.36
**H4ac**	All	−0.041	0.89	−0.081	0.77	0.408	0.13	−0.313	0.30	0.175	0.57	0.252	0.41	−0.125	0.62	0.078	0.76	−0.029	0.91
	Base	−0.547	0.26	−0.458	0.36	0.107	0.84	0.194	0.71	−0.556	0.25	−0.531	0.28	0.188	0.66	0.432	0.29	−0.234	0.58
	Act	0.562	0.12	−0.033	0.93	0.434	0.24	−0.111	0.81	0.349	0.44	0.294	0.52	−0.264	0.46	−0.122	0.74	0.142	0.70
**HDAC2**	All	−0.180	0.52	−0.116	0.68	0.444	0.10	0.262	0.39	***−0.555***	***0.05***	***0.697***	***<0.01***	−0.119	0.64	−0.074	0.77	0.308	0.21
	Base	−0.729	0.10	−0.132	0.80	0.507	0.30	0.059	0.91	***−0.870***	***0.02***	0.695	0.13	0.371	0.37	0.363	0.38	0.431	0.29
	Act	−0.431	0.25	−0.369	0.33	0.472	0.20	−0.629	0.13	***−0.793***	***0.03***	0.507	0.25	−0.412	0.24	−0.309	0.39	0.201	0.58
**pHDAC1**	All	***0.694***	***<0.01***	0.331	0.23	0.143	0.61	0.201	0.51	0.226	0.46	***0.595***	***0.03***	0.078	0.76	−0.361	0.14	−0.116	0.66
	Base	***0.882***	***0.02***	−0.014	0.98	−0.550	0.26	−0.285	0.58	−0.686	0.13	−0.587	0.22	0.452	0.26	−0.662	0.07	−0.298	0.47
	Act	0.608	0.08	−0.137	0.73	−0.336	0.38	−0.564	0.19	***−0.943***	***<0.01***	0.142	0.76	−0.313	0.38	−0.597	0.07	−0.394	0.29
**CBP**	All	0.220	0.43	0.266	0.34	−0.065	0.83	0.348	0.24	0.444	0.13	***0.585***	***0.05***	0.226	0.37	−0.094	0.71	−0.449	0.07
	Base	−0.274	0.60	−0.362	0.48	***−0.859***	***0.03***	−0.352	0.49	−0.503	0.31	−0.210	0.69	−0.546	0.16	−0.490	0.22	−0.578	0.13
	Act	0.061	0.88	−0.025	0.95	−0.555	0.15	−0.058	0.90	0.103	0.83	0.148	0.78	***0.715***	***0.02***	−0.169	0.64	−0.646	0.06

**Table 1** **.** Pearson r correlation coefficients and associated p-values for the learning index scores from initial behavioral characterization in relation to the chromatin modification quantification in Y, AU and AI groups. Significant correlations (p≤0.05) are bolded and italicized.

**Table 2:** Cognitive aging uncouples coordinated epigenetic regulation from individual differences in spatial memory (group analysis).

Group		All (Y,AU,AI)						Intact (Y,AU)						Aged (AU,AI)					
Region		CA1		CA3		DG		CA1		CA3		DG		CA1		CA3		DG	
		r	p	r	p	r	p	r	p	r	p	r	p	r	p	r	p	r	p
**H3aK9**	All	***0.370***	***0.01***	−0.004	0.98	0.137	0.36	***0.499***	***0.01***	−0.285	0.15	0.008	0.97	0.24	0.19	0.012	0.95	0.103	0.58
	Base	0.394	0.09	0.205	0.39	0.331	0.16	***0.682***	***0.02***	−0.488	0.14	−0.092	0.78	0.186	0.52	0.29	0.32	0.233	0.42
	Act	0.279	0.17	−0.139	0.51	−0.022	0.92	0.064	0.81	−0.308	0.27	0.054	0.84	0.186	0.48	−0.129	0.62	−0.032	0.90
**Hac**	All	−0.008	0.96	−0.087	0.57	−0.204	0.17	***−0.391***	***0.05***	−0.152	0.44	***−0.597***	***<0.01***	−0.125	0.52	−0.057	0.76	−0.222	0.23
	Base	−0.165	0.49	−0.413	0.07	0.028	0.91	−0.144	0.66	−0.002	1.00	−0.402	0.20	−0.294	0.31	−0.468	0.09	−0.038	0.90
	Act	***0.407***	***0.05***	0.216	0.29	−0.094	0.65	−0.17	0.56	0.454	0.08	−0.335	0.21	0.137	0.63	0.194	0.46	−0.204	0.43
**H3pS10**	All	***−0.341***	***0.02***	−0.105	0.49	0.068	0.65	***−0.375***	***0.05***	***−0.435***	***0.02***	0.081	0.68	−0.324	0.08	−0.138	0.46	0.087	0.64
	Base	***−0.561***	***0.01***	0.121	0.62	0.216	0.36	***−0.6***	***0.04***	−0.488	0.13	0.215	0.50	−0.355	0.21	0.171	0.56	−0.074	0.80
	Act	−0.244	0.23	−0.185	0.37	−0.026	0.90	−0.445	0.09	−0.333	0.21	0.054	0.84	−0.289	0.26	−0.277	0.28	0.109	0.68
**H4ac**	All	***0.35***	***0.02***	***0.426***	***<0.01***	***0.415***	***<0.01***	−0.039	0.84	0.126	0.52	0.269	0.17	0.028	0.88	0.152	0.41	0.027	0.43
	Base	0.43	0.06	***0.484***	***0.03***	0.306	0.19	0.14	0.67	−0.005	0.99	0.135	0.68	0.104	0.72	0.204	0.48	−0.142	0.63
	Act	***0.478***	***0.01***	0.374	0.06	***0.472***	***0.02***	0.15	0.58	0.108	0.69	0.136	0.62	0.136	0.60	0.091	0.73	0.045	0.86
**HDAC2**	All	−0.139	0.36	***−0.29***	***0.05***	0.159	0.29	0.019	0.92	−0.325	0.09	***0.532***	***<0.01***	−0.12	0.52	***−0.35***	***0.05***	0.213	0.25
	Base	−0.395	0.09	−0.388	0.15	−0.108	0.65	−0.321	0.31	−0.35	0.26	0.542	0.07	−0.328	0.25	−0.28	0.33	−0.172	0.56
	Act	−0.253	0.21	−0.317	0.11	0.247	0.22	−0.482	0.06	***−0.546***	***0.03***	0.454	0.08	−0.212	0.41	−0.39	0.12	0.467	0.06
**pHDAC1**	All	0.268	0.07	0.076	0.62	0.141	0.36	***0.477***	***0.01***	0.292	0.13	***0.377***	***0.05***	0.166	0.37	−0.031	0.87	0.039	0.84
	Base	0.401	0.08	−0.28	0.23	−0.241	0.31	0.383	0.22	−0.256	0.42	−0.265	0.41	0.394	0.16	−0.275	0.34	−0.511	0.06
	Act	0.017	0.93	−0.066	0.75	0.058	0.78	0.08	0.77	−0.469	0.07	−0.177	0.51	−0.163	0.53	−0.239	0.36	0.047	0.86
**CBP**	All	***0.304***	***0.04***	0.143	0.34	−0.043	0.79	0.281	0.15	0.355	0.06	0.222	0.28	0.285	0.12	0.065	0.73	−0.043	0.83
	Base	0.016	0.95	−0.301	0.20	***−0.459***	***0.04***	−0.245	0.44	−0.31	0.33	***−0.581***	***0.05***	−0.049	0.87	−0.362	0.20	−0.431	0.12
	Act	0.365	0.07	0.303	0.13	−0.122	0.58	0.008	0.98	0.039	0.89	−0.36	0.21	0.402	0.11	0.223	0.39	−0.032	0.91

**Table 2** **.** Pearson r correlation coefficients and associated p-values for the learning index scores from initial behavioral characterization in relation to the chromatin modification quantification in collapsed groups: All (Y, AU and AI), cognitively Intact (Y and AU), and Aged (AU and AI). Significant correlations (p≤0.05) are bolded and italicized.

## Discussion

Our results advance the emerging field of cognitive neuroepigenetics on several fronts. Whereas recent studies examining memory have emphasized the significance of chromatin regulation permissive for gene expression, our findings suggest that experience is likely to engage a complex, temporally dynamic pattern of bidirectional modification. Identifying the precise time-course of experience-dependent regulation across multiple epigenetic mechanisms is an important direction in this regard. Our findings indicate that the presence and direction of training induced chromatin modifications are regionally specific across the principal cell fields of the hippocampus. Together with evidence that information is sparsely coded in the hippocampus, this outcome predicts that higher resolution approaches are likely to reveal regulation with greater regional and cellular specificity than currently appreciated. Finally, with respect to our primary focus on cognitive aging, the results demonstrate that the multivariate epigenetic regulation observed in young subjects in relation to hippocampal memory is severely disrupted in aged rats with memory impairment. In another prominent area of investigation on experience-dependent plasticity, current perspectives emphasize the enormous diversity of modification mechanisms and functional roles reflected by experimental phenomena such as long-term potentiation and depression [42]. Our findings may point to an analogous advance, suggesting that the epigenetic control of gene expression critical for normal memory extends beyond permissive and repressive influences, requiring coordinated orchestration among multiple chromatin modifications.

In agreement with previous reports [2], [4], [6], [43], our results revealed reliable experience-dependent regulation of histone acetylation in the hippocampus. A majority of earlier work has utilized contextual fear conditioning as a setting for exploring epigenetic contributions to memory, and the present findings confirm that other canonical tests of hippocampal function can also engage chromatin modification dynamics (see also [2]). In this case, however, the pattern was more complex, involving not only hippocampus-wide regulation, but also subfield- and lysine-specific responses, and both increased and decreased experience-dependent histone acetylation. Specifically, while histone H3 acetylation was potently induced following water maze training across all regions of the hippocampus, acetylation of lysine 9 on H3 was downregulated selectively in CA1. Although there is precedent that experience can differentially regulate H3 and H4 acetylation [4], here we document that this effect is dependent on hippocampal subregion such that H4 acetylation is influenced in opposite directions in CA1 and DG, and insensitive to recent experience in CA3. This previously unrecognized regional selectivity suggests that results from homogenates of whole hippocampus, indicating that learning is uniformly associated with increased histone acetylation, may reflect an aggregate, averaged response masking finer grain, circuit-level regulation.

Specificity in the epigenetic control of gene expression critical for memory is thought to arise from the coordinated regulation of multiple modifications. Fidelity in deciphering these combinatorial codes is dependent on the specificity of available reagents, and in the present experiments, we confirmed by standard competition assays that incubation with excess peptide eliminates signal at the appropriate molecular weight for all histone and HDAC primary antibodies used in the analysis (see S1). Taking advantage of newly developed peptide array technology [44], however, we were surprised that many of the histone antibodies tested recognize multiple modifications in addition their intended target. Antibodies against H3aK9 and H3pS10 proved largely selective (Fig. S4A, C). In contrast, while the antibody used to quantify H3a recognized multiple acetylated lysine sites on H3 (Fig. S4B), it is commercially supplied as H3-acetylK14 selective. Similarly, peptide array analysis revealed that the antibody we used to document age and experience-dependent effects on hippocampal histone H4 acetylation (H4a) recognizes multiple H4 acetylation sites in addition to the supplier-intended K12 modification (Fig. S4D). The latter observation is noteworthy in that it may be among the factors contributing to the apparent discrepancy between our results and those of Peleg *et al.* [5], where deficits in fear conditioning in aged mice were reportedly associated with a loss of experience-dependent histone acetylation selectively at H4K12. The broader implication, amplified elsewhere [45], is that comparisons among neuroepigenetic studies are significantly compromised in the absence of evidence explicitly documenting antibody cross-reactivity.

Aged rats in our experiments exhibited increased histone H4 acetylation relative to young adults across all hippocampal subfields, independent of cognitive status and recent behavioral experience. These findings count against the thrust of a current literature suggesting that elevated histone acetylation levels broadly benefit memory. The constitutive increase in acetylation we observed with aging also sounds a cautionary note with respect to the prediction that inducing chronic hyperacetylation by HDAC inhibitor administration might rescue age-related cognitive impairment [7], [33], [38]. Our data encourage the view that the role of epigenetic regulation in normal and impaired cognitive function is likely more nuanced, involving the dynamic orchestration and interactive influence of multiple modifications. Consistent with this notion, we failed to detect any single epigenetic mark uniquely associated with age-related impairment mediated by the hippocampus. Spatial memory capacity was instead coupled with a broad array of multiple chromatin modification factors in both baseline and behaviorally tested young and aged unimpaired rats, including instances from all histone PTMs examined, and both HAT and HDAC levels (Tables 1,2). Such coupling was entirely absent in the hippocampus of aged rats with documented memory deficits, suggesting that impairment may arise in part from a disruption in broad scale coordination across the epigenetic landscape. The significant implication for the development therapeutic interventions is that effective strategies may hinge on restoring coordinated control, rather than simply promoting up- or downregulation.

Experience-dependent histone acetylation reflects the operation of multiple regulatory processes, and our analysis included the key mediators of bidirectional control, HDACs and HATs. Recent attention has focused on class I HDACs, specifically HDAC1 and 2, with current evidence pointing to a critical and selective role for HDAC2 in memory related synaptic plasticity [7]. The notable finding reported here is that HDAC2 protein was significantly decreased following behavioral training in the CA1 field of the young hippocampus, and while this response appears intact in aged rats with preserved memory, the hippocampus in AI rats failed to exhibit reliable regulation. Experience-dependent downregulation of HDAC2 was also observed in AU rats, selectively in the dentate gyrus, raising the possibility that this response reflects a successful epigenetic adaptation. We also examined levels of CBP, i.e., a transcriptional co-activator with intrinsic HAT activity that is necessary for normal learning and memory [9]. Consistent with recent findings from Bousiges *et al.* [2], CBP was potently regulated in response to recent behavioral experience across all hippocampal subfields. This effect, however, was unrelated to chronological age or cognitive status. Together, these findings underscore the importance of considering the dynamic regulation of chromatin modifying enzymes alongside resulting changes in histone acetylation in the study of epigenetic contributions to memory. Identifying the temporal dynamics of the interacting processes that mediate, circuit-specific, experience-dependent chromatin regulation remains a key challenge for investigation.

## Materials and Methods

### Subjects

A total of 71 specific pathogen-free male Long-Evans rats (Charles River Laboratories) were housed singly in a climate-controlled vivarium on a 12∶12 hour light∶dark cycle. Animals with frank pathologies expected to influence the principal outcome measures were excluded. Standard lab chow and water were available ad libitum. All animals were behaviorally characterized in the Morris water maze in order to document the status of hippocampus dependent spatial learning (see detailed methods, below). A subset (9 Y, 7 AU, 10 AI) was also trained one month later on a redundant place-cue (RPC) water maze procedure as an induction event for examining the influence of recent experience on chromatin modification. These results were compared with resting values from age- and cognitive status-matched rats (6 Y, 6 AU, 8 AI) sacrificed directly from the home cage a minimum of two weeks after standard water maze testing. RPC training was also omitted for an additional set of nine animals (3 Y, 3 AU, 3 AI), and brains from these rats were used to determine HDAC cellular localization by immunocytochemical labeling and confocal microscopy. Finally, a cohort of young rats (8 home cage and 8 RPC-trained) was used to examine by immunocytochemical visualization the effect of recent behavioral experience on phosphorylation of histone H3 serine 10.

### Background Behavioral Characterization

Male Long-Evans rats, 6 or 24 months of age, were behaviorally characterized in a standardized place version of the Morris water maze, as described in previous studies [29]. Briefly, training was conducted over 8 consecutive days, each consisting of three 90 s trials with a 60 s inter-trial interval. An escape platform, hidden below the surface of the water, was maintained in a constant location throughout training and start locations were varied pseudo-randomly. A probe trial, in which the platform was retracted to the bottom of the maze for 30 s and then raised to allow escape, was provided every sixth trial (i.e., the last trial of every other day). Performance was assessed using a learning index score (a weighted average proximity (cm) to the escape location over the course of probe trials). Based on this index, aged rats were classified as impaired (scores above 240) or unimpaired (scores below 240). Animals were also tested on a non-spatial, cued platform version of the water maze in a single session of six trials, to evaluate sensorimotor function, motivation to escape, and other general performance factors. Animals that fail this hippocampus-independent task variant are excluded from further analysis.

### Redundant Place-Cue (RPC) Task

The inducing event used to examine experience-dependent chromatin regulation consisted of a redundant place-cue procedure adapted form earlier work [32]. In this task a representative subset of the young and aged rats that received standard water maze training (described above) were tested one month later in a modified, 1-session protocol that allowed for both hippocampus dependent and independent solutions (9 Y; 7 AU; 10 AI). Testing was conducted in a different room than prior water maze training. The RPC task consisted of 17 trials with a 15 s inter-trial interval, initiated 2 hr before sacrifice. A 60-second probe trial, in which the platform was retracted to the bottom of the pool, was provided immediately before sacrifice. For trials 1 through 9, and 11, 13, 15, and 17, the escape platform was cued by a black cap that protruded 3 cm above the water surface. On the remaining four, interleaved trials (10, 12, 14, and 16), the cap was removed, leaving the platform slightly submerged and hidden. The escape platform remained in a constant location throughout training, and the start location was varied pseudo-randomly across trials. Performance was measured by pathlength (total swim distance from the start to the platform) during acquisition, and proximity (average distance from the escape location) during probe testing. Two hours after the first training trial and immediately following the probe trial, animals were rapidly anesthetized with 5% isoflurane, decapitated, and brains were extracted. The dentate gyrus, CA1, and CA3 subregions were microdissected on ice under a stereoscope, and samples were frozen at −80°C until further processing.

### Ethics Statement

This study was carried out in strict accordance with the recommendations in the Guide for the Care and Use of Laboratory Animals of the National Institutes of Health. The protocol was approved by the Animal Care and Use Committee of the National Institute on Aging (ASP Number 407-LEG-2012). All efforts were made to minimize suffering.

### Subcellular Fractionation

Microdissected hippocampi were homogenized in a hypotonic buffer (10 mM HEPES, 1.5 mM MgCl_2_, 10 mM KCl, and 2× Protease Inhibitor; Thermo Scientific). Samples were then incubated on ice for 30 min and subsequently lysed with a syringe. After 15 min incubation on ice, samples were centrifuged at 1,000 g at 4°C for 15 min. The supernatant (cytosolic fraction) was saved and made into a 2% SDS solution and the nuclear pellet was washed with hypotonic buffer and subsequently centrifuged at 1,000 g at 4°C for 15 min. The pellet was resuspended in 5% SDS and stored at −80°C. All immunoblots utilized the nuclear fractions.

### Immunoblotting

Samples (15 Y, 13 AU, 18 AI) were normalized for total protein concentration using a protein assay kit (Thermo Scientific) and separated by SDS-PAGE (NuPage 3–8% Tris-Acetate or 12% Bis-Tris; Invitrogen). Primary antibodies to histones H3-acetyl-K9 (1∶1000; Abcam ab4441), H3-acetyl-K14 (1∶1000; Abcam ab52946; here termed H3-pan-acetyl (H3a) on the basis of peptide array findings), H3-phospo-S10 (1∶1000; Abcam ab5176), H4-acetyl-K12 (1∶250; Abcam ab46983; here termed H4-pan-acetyl (H4a) on the basis of peptide array findings), H3-pan (1∶2500; Abcam ab10799), HDAC1-phospho-S421 (1∶1000; Abcam ab63884), CBP (1∶200; Santa Cruz Biotechnology sc-583), HDAC1 (1∶500; Biovision 3601-100), HDAC2 (1∶500; Biovision 3602-100), and β-actin (1∶1000; Biovision 3662-100) were diluted in blocking solution (2% ECL advance blocking agent; GE Healthcare) in wash buffer (phosphate buffered saline with 0.1% Tween-20) and applied to membranes overnight at 4°C. β-actin or H3-pan was used as a protein loading control. Immunoreactivity was detected with Alexa 488 and Alexa 633 conjugated secondary antibodies (1∶2500; Invitrogen). All groups and conditions were represented within each blot for each hippocampal subfield. Immunoblots were scanned at a resolution of 100 µm/pixel on a Typhoon Trio Plus Scanner (GE Healthcare) and bands were analyzed using ImageQuant TL image analysis software (GE Healthcare). Competition assays using excess peptide completely eliminated signal at the appropriate MW for all histone and HDAC antibodies. In addition to peptide competition assays, all histone antibodies were tested for site- and modification-specificity using a histone peptide array (Active Motif). Whereas some antisera proved highly selective (H3aK9 and H3pS10), others (i.e., H3-acetylK14 and H4-acetylK12) recognized multiple histone modifications in addition to their intended target (see results, and Figure S4).

### Immunocytochemistry

Animals were deeply anesthetized with 5% isoflurane and perfused transcardially with ∼105 ml of cold 0.1 M phosphate buffered saline (PBS), followed by ∼450 ml of cold 4% paraformaldehyde in 0.1 M PBS (pH 7.2–7.4), at a rate of 35 ml/min. Brains were removed and post-fixed overnight in perfusate at 4°C. This was followed by two successive overnight incubations at 4°C in cryoprotectant (10% followed by 20% glycerol in 0.1 M phosphate buffer). Tissue was stored at −80°C until sectioning on a freezing/sliding microtome. Fifty-micrometer sections were taken through the rostro-caudal extent of the hippocampus. Sections were stored in 30% glycerol and 30% ethylene glycol in 0.1 M PB at −80°C. Immunohistochemical procedures utilized 1× PBS and PBST (PBS+1.0% Triton-X) washes. For studies examining HDAC and histone PTM localization, approximately 5 sections per brain from the dorsal hippocampus (spaced 500 µm apart) were incubated with the following polyclonal primary anti-sera overnight at 4°C: anti-HDAC1 (1∶1000; Abcam), anti-GFAP (1∶1000; Abcam), and anti-HDAC2 (1∶200; Biovision), H3-acetyl-K9 (1∶1000; Millipore), Histone-pan-acetyl (1∶500; Millipore), H3-phospo-S10 (1∶500; Millipore), and H4-pan-acetyl (1∶10000; Millipore). Sections were subsequently incubated with AlexaFluor secondary antibodies (1∶200; Invitrogen) for 1 hour. Slides were coverslipped with PVA-DAPCO (Sigma) or ProLong Gold (Invitrogen). Digital images were acquired on a Zeiss LSM 710 confocal microscope. Stereological quantification of H3-phosphoS10 immunopositive cells in the top and bottom blade of the DG was conducted using Stereo Investigator software (MicroBrightfield, Inc).

### Antibody Specificity Analysis Using Peptide Array Technology

In addition to peptide competition assays, all histone antibodies were tested for site- and modification-specificity using a histone peptide array (Active Motif) comprised of 384 differentially modified histone tail variants. Specificity factors were calculated using Active Motif software. Of note, whereas all antibodies bound their intended targets, H3-acetylK14 and H4-acetylK12 labeled additional modifications.

### Statistical Analysis

Parametric statistics (ANOVA) were used to compare relevant behavioral outcome measures, histone PTMs, HDAC's, and CBP levels across age, cognitive status, and training conditions. Pearson r correlation coefficients were used to assess correlations in relation to individual differences in spatial learning and memory capacity.

## Supporting Information

Figure S1 **H3-phosphoS10 immunoreactivity is restricted and distinct from histone acetylation PTMs.** (A) Digital photomicrographs of immunocytochemical labeling of H3aK9, Histone-pan-acetyl, H3pS10, and H4-pan-acetyl in the hippocampus. Note the distinct localization pattern of H3pS10 among a small subset of dentate gyrus granule cell neurons. (B) Stereological quantification of H3pS10 immunopositive cells in the top and bottom blades of the dentate gyrus granule cell layer in young animals after RPC training, and corresponding representative images of immunocytochemical staining. Asterisks indicate p<0.05 relative to baseline.
(TIF)

Figure S2 **Phospho-S421 HDAC1 and CBP are robustly regulated by recent behavioral experience.** (A) Quantification of phospho-S421 HDAC1 levels in region CA1 (top), CA3 (middle), and DG (bottom). Representative western blots are organized according to the corresponding quantitative results. (B) Quantification of CBP levels and accompanying representative western blots in region CA1 (top), CA3 (middle), and DG (bottom). Asterisks denote statistically significant (p<0.05) comparisons between subjects with (Activated) or without (Baseline) RPC training. Error bars = S.E.M.
(TIF)

Figure S3 **Differential cellular localization of HDAC1 and 2 immunoreactivity in the hippocampus.** (A) Representative single channel and merged confocal microscope images of HDAC2, HDAC1 and GFAP immunofluorescence staining in hippocampal subfields of Y, AU and AI rats. HDAC2 staining is predominantly localized to neuronal nuclei, whereas HDAC1 immunoreactivity is relatively enriched among GFAP-positive astrocytes. Note the near absence of HDAC1 labeling in CA3 pyramidal neurons. Substantial non-nuclear HDAC1 signal was also detected. (B) Tiled composite confocal image of the hippocampus displaying localization patterns of HDAC1 (green), HDAC2 (red), and GFAP (blue).
(TIF)

Figure S4 **Peptide array technology (Active Motif) reveals the scope of histone PTM antibody selectivity.** (A–D) Specificity value, calculated as the ratio of positively identified site modifications to negative site modifications, of various commercially available antibodies: (A) H3acetyl-K9, (B) H3acetyl-K14 (here termed H3-pan-acetyl (H3a) for western blotting methodology, where the band at the appropriate molecular weight for H3 was quantified, and termed Histone-pan-acetyl (Ha) for immunocytochemistry in Figure S1), (C) H3pS10, and (D) H4acetly-K12 (here termed H4-pan-acetyl, H4a). (E) Peptide array technology provides an evaluation of both antibody specificity and steric hindrance. The H3pS10 antibody, for example, was selective for the intended target (blue circled blots) and did not recognize most modifications in the absence of phospho-serine-10 (e.g., H3-acetylK9; red, and H3-phosphoT11; yellow). In the presence of phosphoT11, however, the antibody failed to bind phospho-S10 (green circles).
(TIF)
