# Supplementary figures and images for: Age-Related Memory Impairment Is Associated with Disrupted Multivariate Epigenetic Coordination in the Hippocampus
Source: PLoS One. 2012 Mar 15;7(3):e33249. doi: 10.1371/journal.pone.0033249 (PMC3305324; doi:10.1371/journal.pone.0033249)

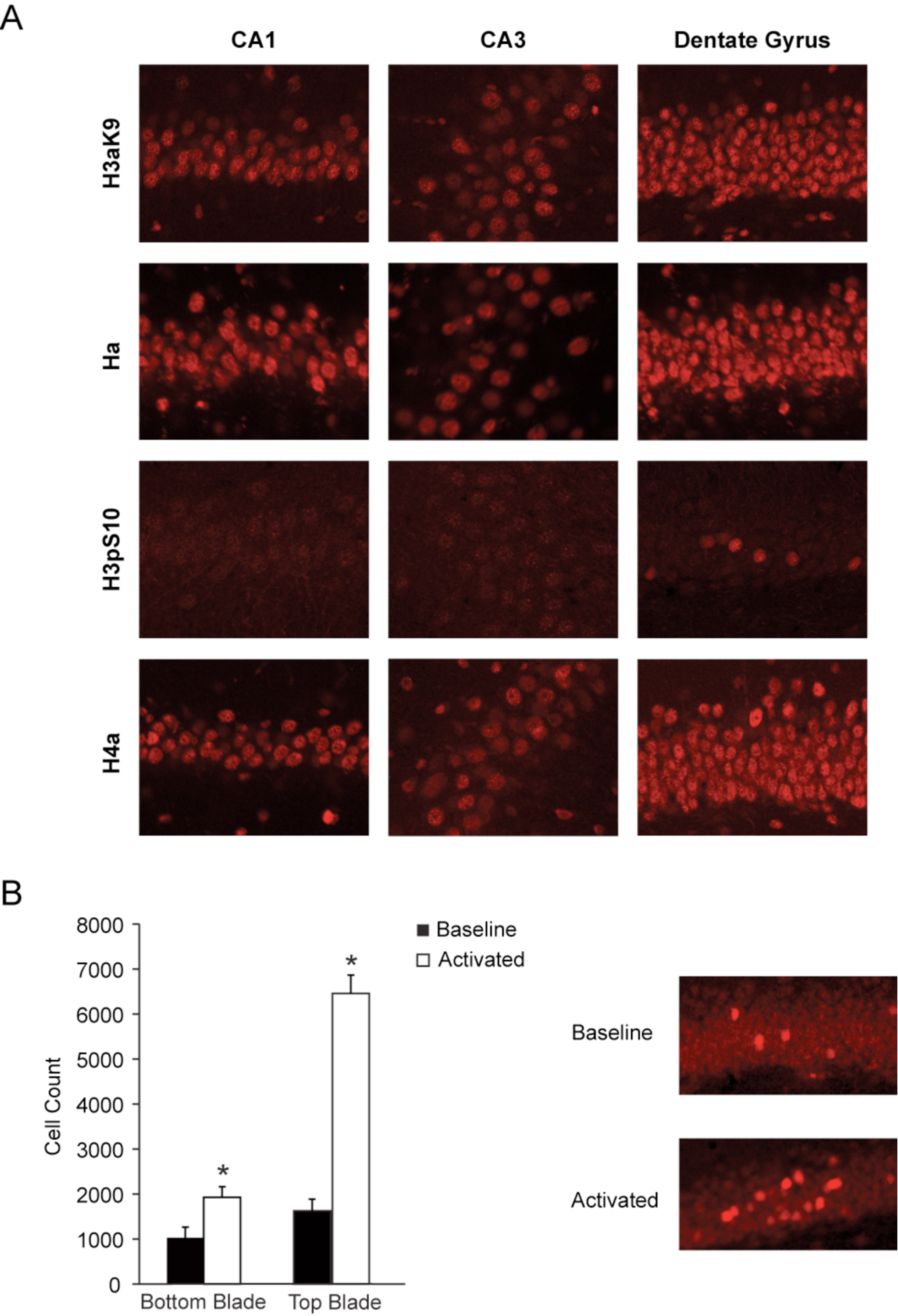

Supplement: Figure S1 — H3-phosphoS10 immunoreactivity is restricted and distinct from histone acetylation PTMs. (A) Digital photomicrographs of immunocytochemical labeling of H3aK9, Histone-pan-acetyl, H3pS10, and H4-pan-acetyl in the hippocampus. Note the distinct localization pattern of H3pS10 among a small subset of dentate gyrus granule cell neurons. (B) Stereological quantification of H3pS10 immunopositive cells in the top and bottom blades of the dentate gyrus granule cell layer in young animals after RPC training, and corresponding representative images of immunocytochemical staining. Asterisks indicate p<0.05 relative to baseline. (TIF) [file pone.0033249.s001.tif]

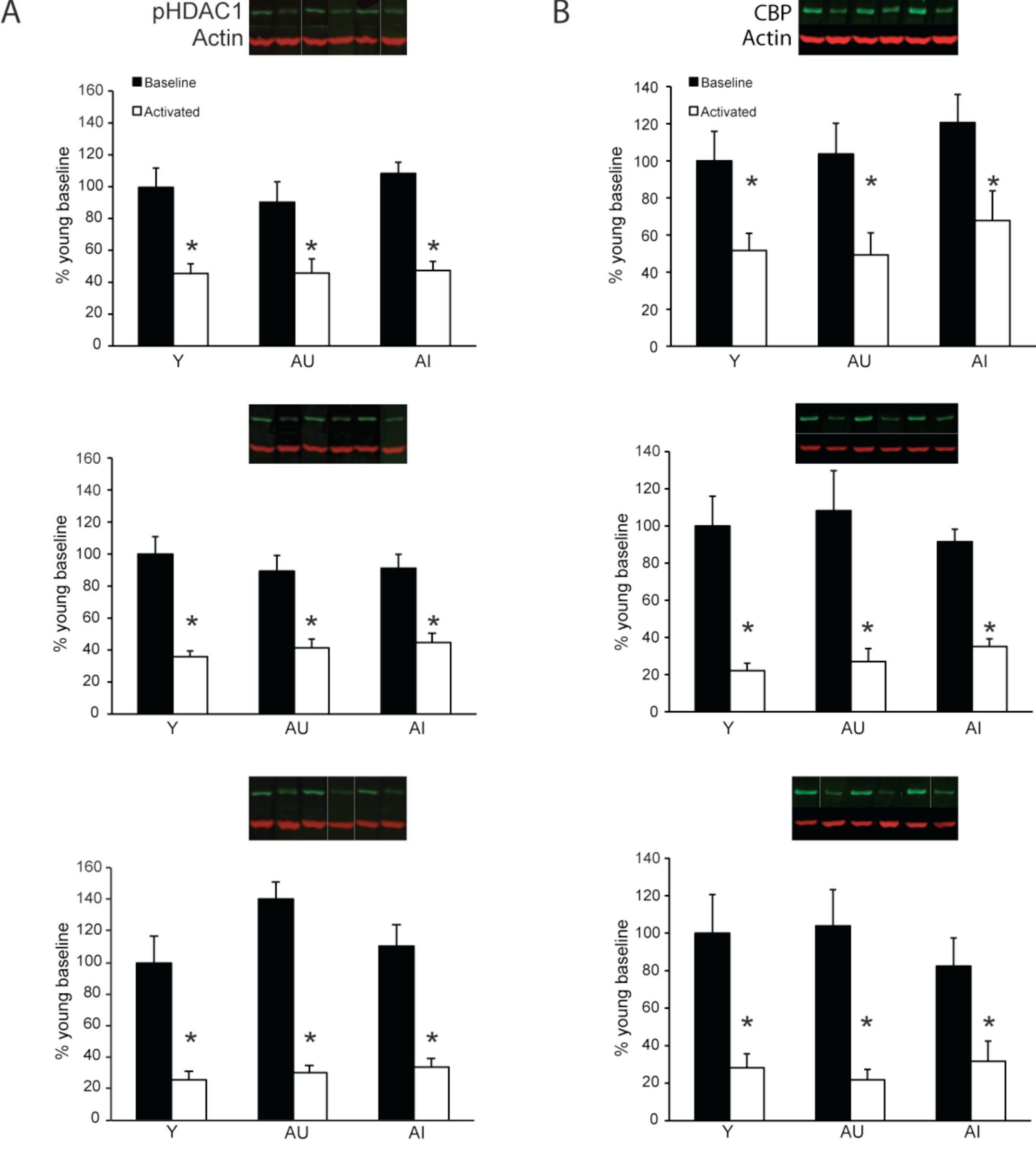

Supplement: Figure S2 — Phospho-S421 HDAC1 and CBP are robustly regulated by recent behavioral experience. (A) Quantification of phospho-S421 HDAC1 levels in region CA1 (top), CA3 (middle), and DG (bottom). Representative western blots are organized according to the corresponding quantitative results. (B) Quantification of CBP levels and accompanying representative western blots in region CA1 (top), CA3 (middle), and DG (bottom). Asterisks denote statistically significant (p<0.05) comparisons between subjects with (Activated) or without (Baseline) RPC training. Error bars = S.E.M. (TIF) [file pone.0033249.s002.tif]

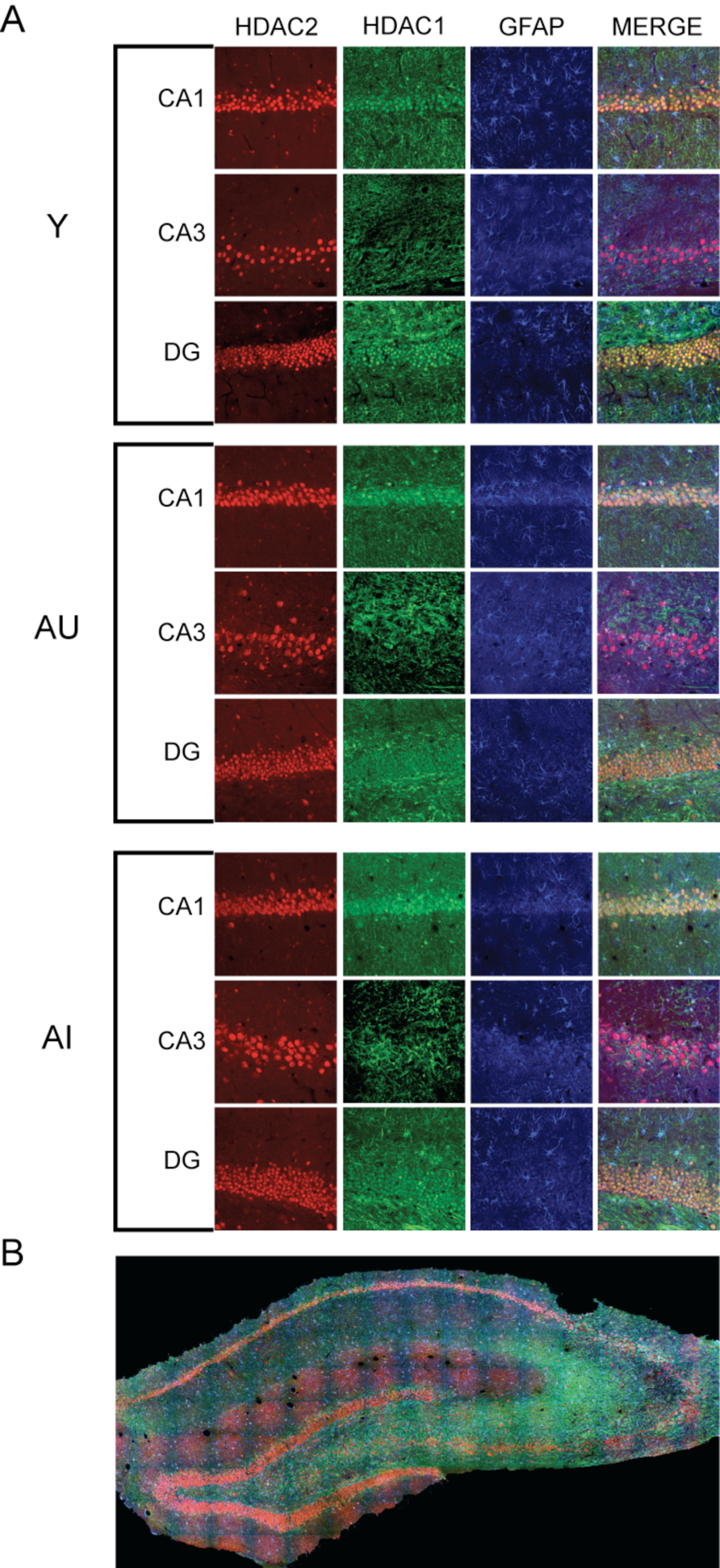

Supplement: Figure S3 — Differential cellular localization of HDAC1 and 2 immunoreactivity in the hippocampus. (A) Representative single channel and merged confocal microscope images of HDAC2, HDAC1 and GFAP immunofluorescence staining in hippocampal subfields of Y, AU and AI rats. HDAC2 staining is predominantly localized to neuronal nuclei, whereas HDAC1 immunoreactivity is relatively enriched among GFAP-positive astrocytes. Note the near absence of HDAC1 labeling in CA3 pyramidal neurons. Substantial non-nuclear HDAC1 signal was also detected. (B) Tiled composite confocal image of the hippocampus displaying localization patterns of HDAC1 (green), HDAC2 (red), and GFAP (blue). (TIF) [file pone.0033249.s003.tif]

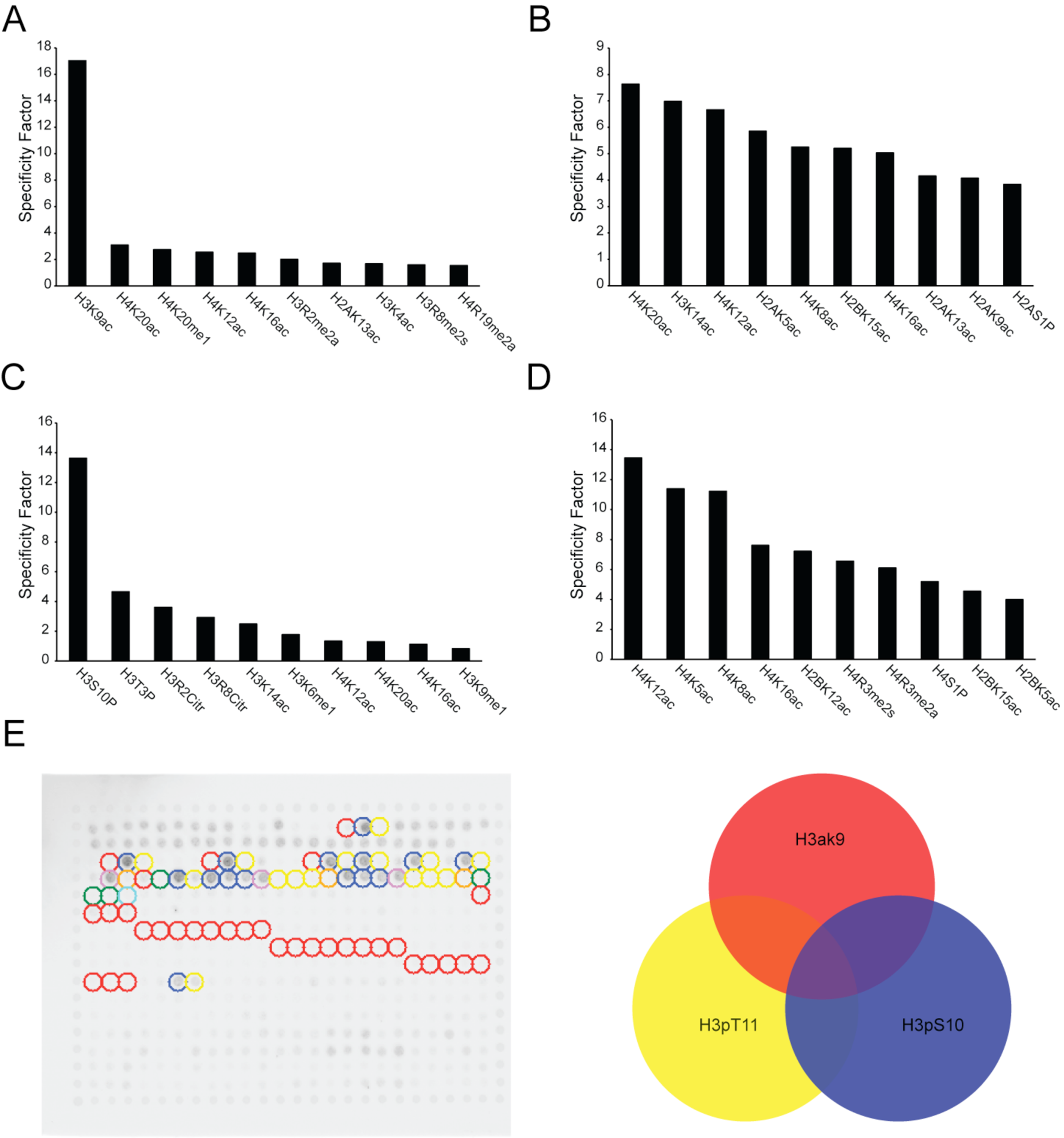

Supplement: Figure S4 — Peptide array technology (Active Motif) reveals the scope of histone PTM antibody selectivity. (A–D) Specificity value, calculated as the ratio of positively identified site modifications to negative site modifications, of various commercially available antibodies: (A) H3acetyl-K9, (B) H3acetyl-K14 (here termed H3-pan-acetyl (H3a) for western blotting methodology, where the band at the appropriate molecular weight for H3 was quantified, and termed Histone-pan-acetyl (Ha) for immunocytochemistry in Figure S1), (C) H3pS10, and (D) H4acetly-K12 (here termed H4-pan-acetyl, H4a). (E) Peptide array technology provides an evaluation of both antibody specificity and steric hindrance. The H3pS10 antibody, for example, was selective for the intended target (blue circled blots) and did not recognize most modifications in the absence of phospho-serine-10 (e.g., H3-acetylK9; red, and H3-phosphoT11; yellow). In the presence of phosphoT11, however, the antibody failed to bind phospho-S10 (green circles). (TIF) [file pone.0033249.s004.tif]
